# Supplementary material for: The tetracycline resistome is shaped by selection for specific resistance mechanisms by each antibiotic generation
Source: Nat Commun. 2025 Feb 7;16:1452. doi: 10.1038/s41467-025-56425-5 (PMC11806011; doi:10.1038/s41467-025-56425-5)
Supplement: Supplementary file 1 — Supplementary Information [file 41467_2025_56425_MOESM1_ESM.pdf]

## Supplementary Information

# The tetracycline resistome is shaped by selection for specific resistance mechanisms by each antibiotic generation

Kevin S. Blake [1,2,†], Yao-Peng Xue [1], Vincent J. Gillespie [1], Skye R.S. Fishbein [1,2], Niraj H. Tolia [3,*] Timothy A. Wencewicz [4,*], Gautam Dantas [1,2,5,6,7,*]

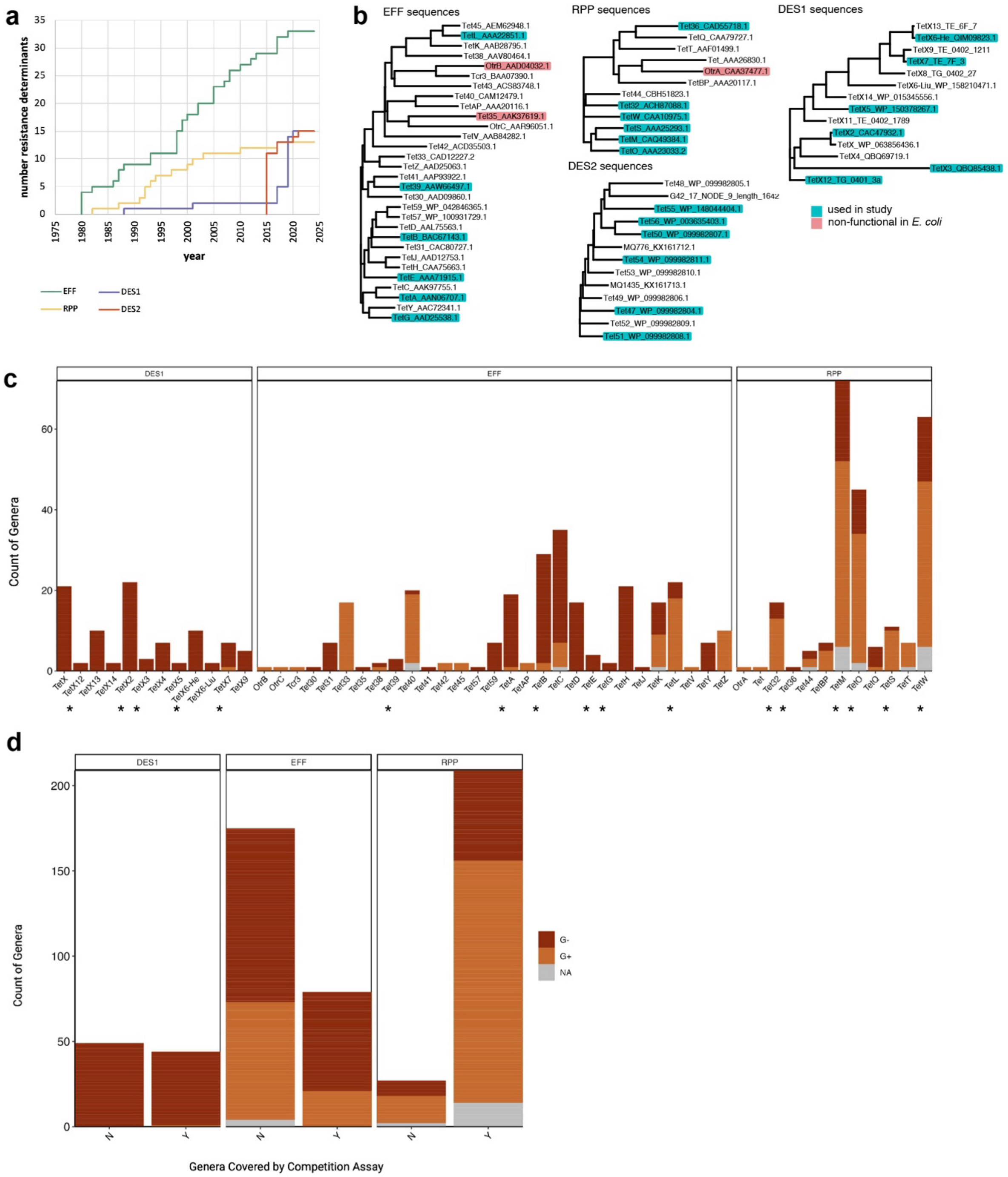


**Supplementary Figure 1. (a)** Count of characterized tetracycline resistant determinants over time. With the caveat that advancements in molecular and sequencing techniques have made functional gene discovery and differentiation easier, and that the criteria for naming resistance determinants differ between fields and have changed over time. **(b)** Neighbor-joining trees of known genes from each mechanism. Tip labels include gene name, and the GenBank accession number of the sequence used. Blue highlight = used in this study. Red highlight = was inserted into *E. coli*

DH5αZ1 + pZE24 system but did not confer resistance greater than empty control. **(c)** Count of unique genera observed by each gene in the tetracycline resistome. Faceted by mechanism, with asterisks denoting genes used in downstream analyses. DES2 genes were not analyzed as only one sequence has been associated with a species' genome (the rest were identified by functional metagenomic selection). **(d)** Count of unique genera by mechanism for the sequences selected for downstream analyses (Y) or not included (N). EFF = efflux pump; RPP = ribosomal protection protein; DES1 = type 1 tetracycline destructase; DES2 = type 2 tetracycline destructase.

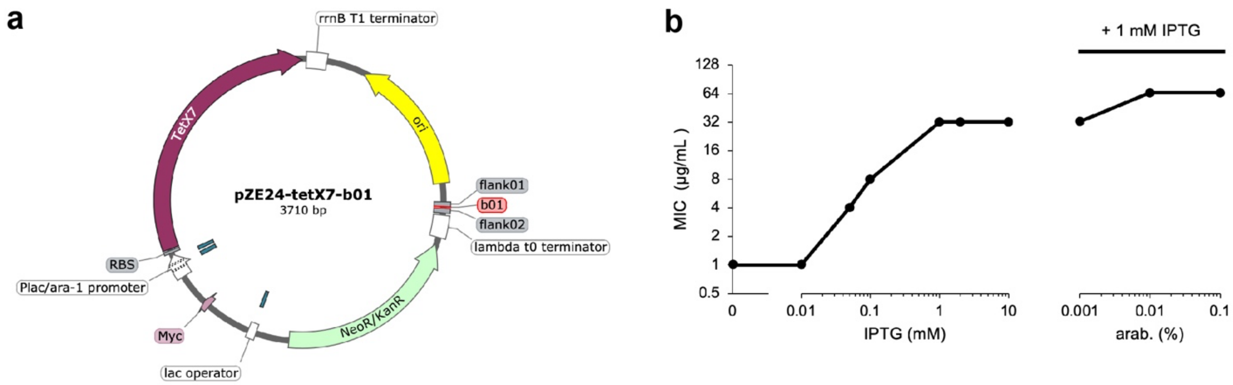


**Supplementary Figure 2. (a)** Construct map of the plasmids used in this study. **(b)** AST with tetracycline was performed for *E. coli* DH5alphaZ1 + pZE24-Tet(56-2) [1] in growth media supplemented with different concentrations of IPTG, and IPTG plus L-arabinose (arab). Similar to protein expression [2], MIC to tetracycline plateaus at 1 mM IPTG, with a second plateau at 1 mM IPTG + 0.01% arabinose.

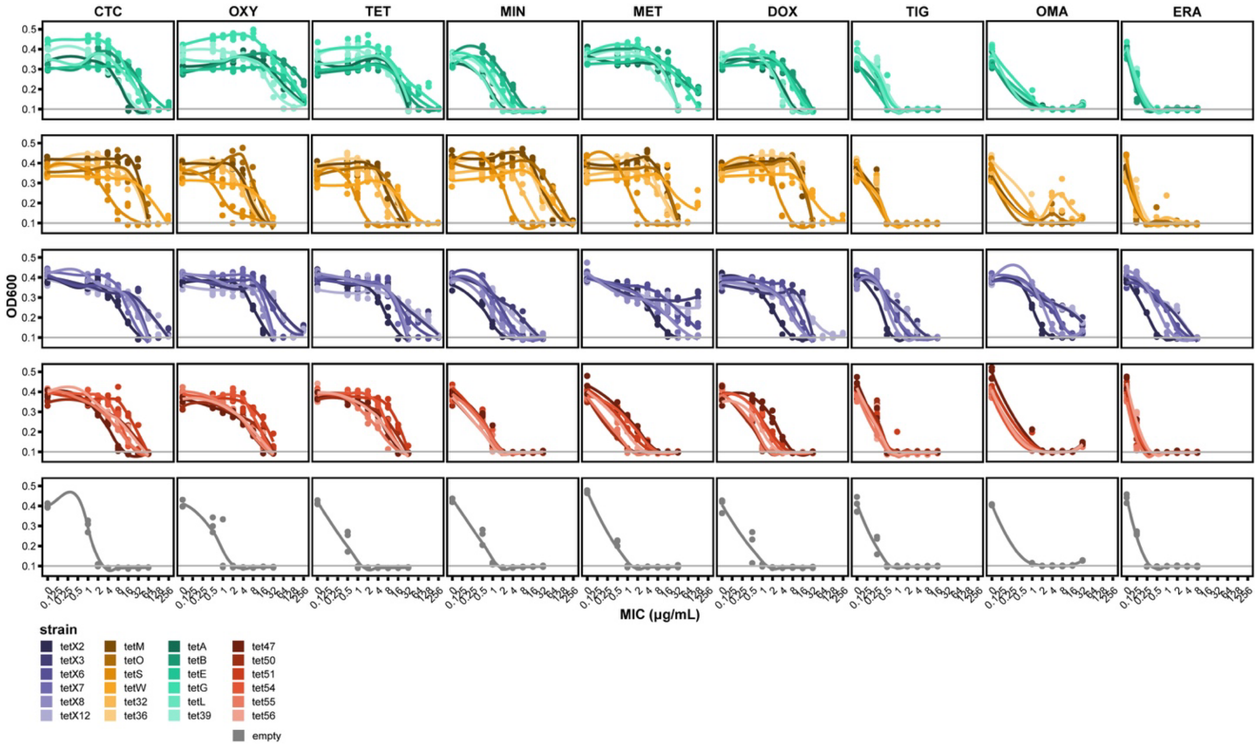


**Supplementary Figure 3.** Dosage curves for each strain in ASTs. Points correspond to the endpoint $OD_{600}$ of each technical replicate for each strain at each drug-concentration combination (3 per strain-drug-concentration combination). Horizontal line corresponds to $OD_{600} = 0.1$, the approximate background $OD_{600}$ of the media and therefore represents no cell growth. Drug-concentrations which increased the background $OD_{600} > 0.1$ were masked. Abbreviations: TET = tetracycline; DOX = doxycycline; CTC = chlortetracycline; DOX = doxycycline; MIN = minocycline; MET = metacycline; EFF = efflux pump; RPP = ribosomal protection protein; DES1 = type 1 tetracycline destructase; DES2 = type 2 tetracycline destructase.

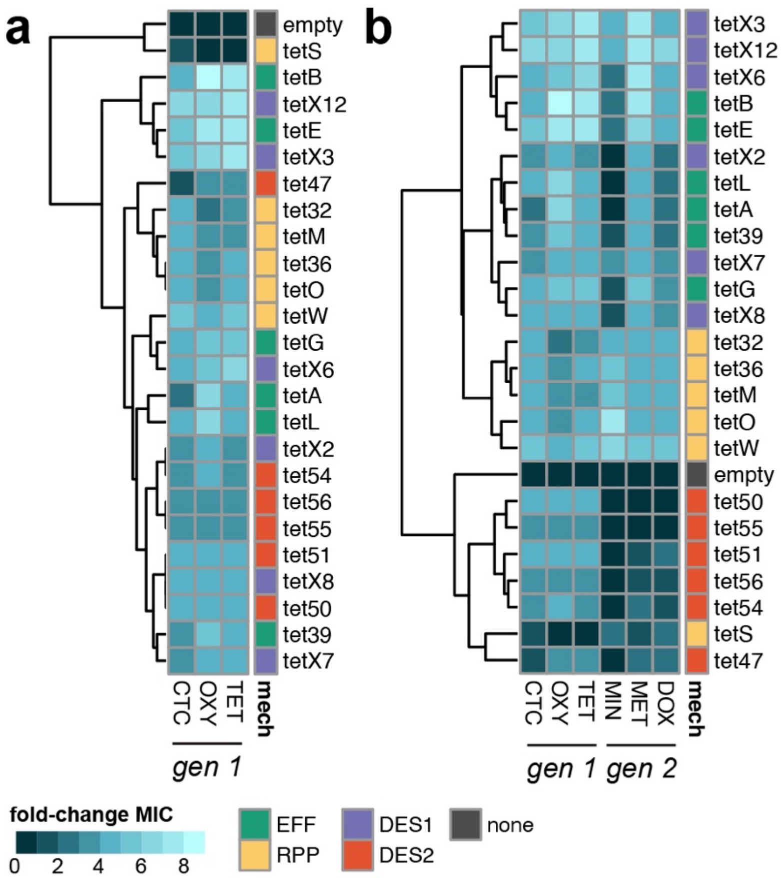


**Supplementary Figure 4.** Heatmap of AST performed for each strain against **(a)** first-generation tetracyclines, and **(b)** second-generation tetracyclines. Data is a subset of Figure 2A. Resulting MIC values for each strain-antibiotic combination plotted as log2 fold-change over the empty vector strain's MIC for that antibiotic. Actual MIC values are listed in Supplementary Data 3. Abbreviations: TET = tetracycline; DOX = doxycycline; CTC = chlortetracycline; DOX = doxycycline; MIN = minocycline; MET = metacycline; EFF = efflux pump; RPP = ribosomal protection protein; DES1 = type 1 tetracycline destructase; DES2 = type 2 tetracycline destructase.

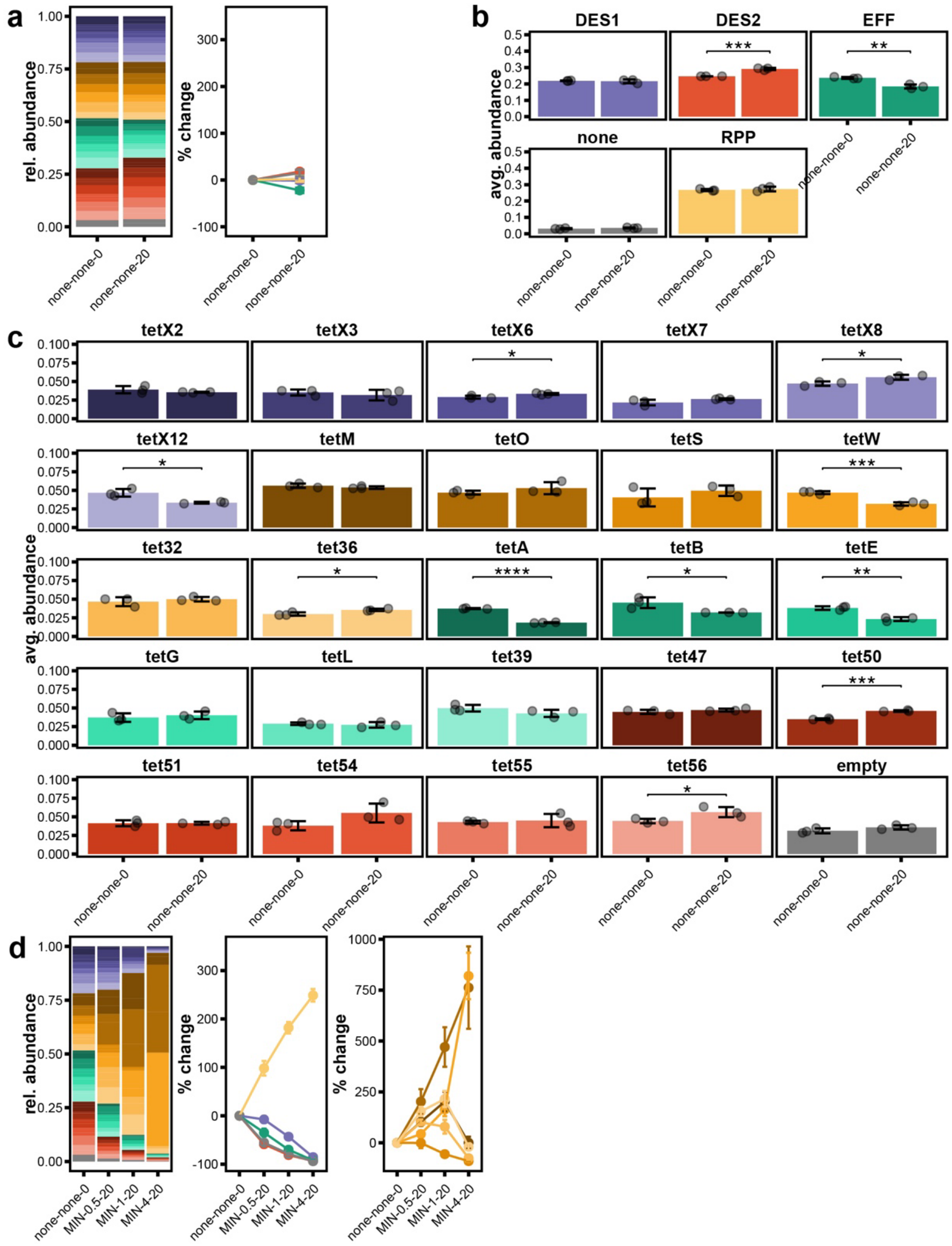


**Supplementary Figure 5. (a)** Results for the mix containing all 25 strains (ALL mix) grown for 20 hours in tetracycline-free media. Left panel: barplots of the relative abundance of each strain in the starting mix, then after growth in the antibiotic selective conditions at 0.5x, 1x, and 4x the MIC of the empty vector strain. Bar height represents the average of three replicates. Right panel: the percent change in the summed relative abundances of each strain belonging to a given mechanism

relative to the starting mixture. Points represent the average of three replicates, and error bars represent standard deviation. **(b)** Comparisons of the mean summed relative abundance of each mechanism between the ALL mix and after 20 hours growth in tetracycline-free media. Bar represents the mean relative abundance of three technical replicates, and error bars indicate the standard deviation. Means compared using pairwise t-test with Bonferroni correction. Points represent each of the three technical replicates. **(c)** Comparisons of the mean relative abundance of each strain between the ALL mix and after 20 hours growth in tetracycline-free media. Bar represents the mean relative abundance of three technical replicates, and error bars indicate the standard deviation. Means compared using pairwise t-test with Bonferroni correction. Points represent each of the three technical replicates. **(d)** Results for the mix containing all 25 strains (ALL mix) grown with 0.5x, 1x, and 4x empty strain MIC in the context of minocycline ($2^{nd}$ gen). X-axis labels denote antibiotic-concentration-timepoint (hours). Left panel: barplots of the relative abundance of each strain in the starting mix, then after growth in the antibiotic selective conditions at 0.5x, 1x, and 4x the MIC of the empty vector strain. Bar height represents the average of three replicates. Middle panel: the percent change in the summed relative abundances of each strain belonging to a given mechanism relative to the starting mixture. Right panel: the percent change in the relative abundance of each strain encoding genes belonging to the dominant mechanism under each antibiotic selective condition. Points represent the average of three replicates, and error bars represent standard deviation. * = $p < 0.05$, ** = $p < 0.005$, *** = $p < 0.0005$, and **** = $p < 0.00005$. Abbreviations: TET = tetracycline; DOX = doxycycline; TIG = tigecycline; EFF = efflux pump; RPP = ribosomal protection protein; DES1 = type 1 tetracycline destructase; DES2 = type 2 tetracycline destructase.

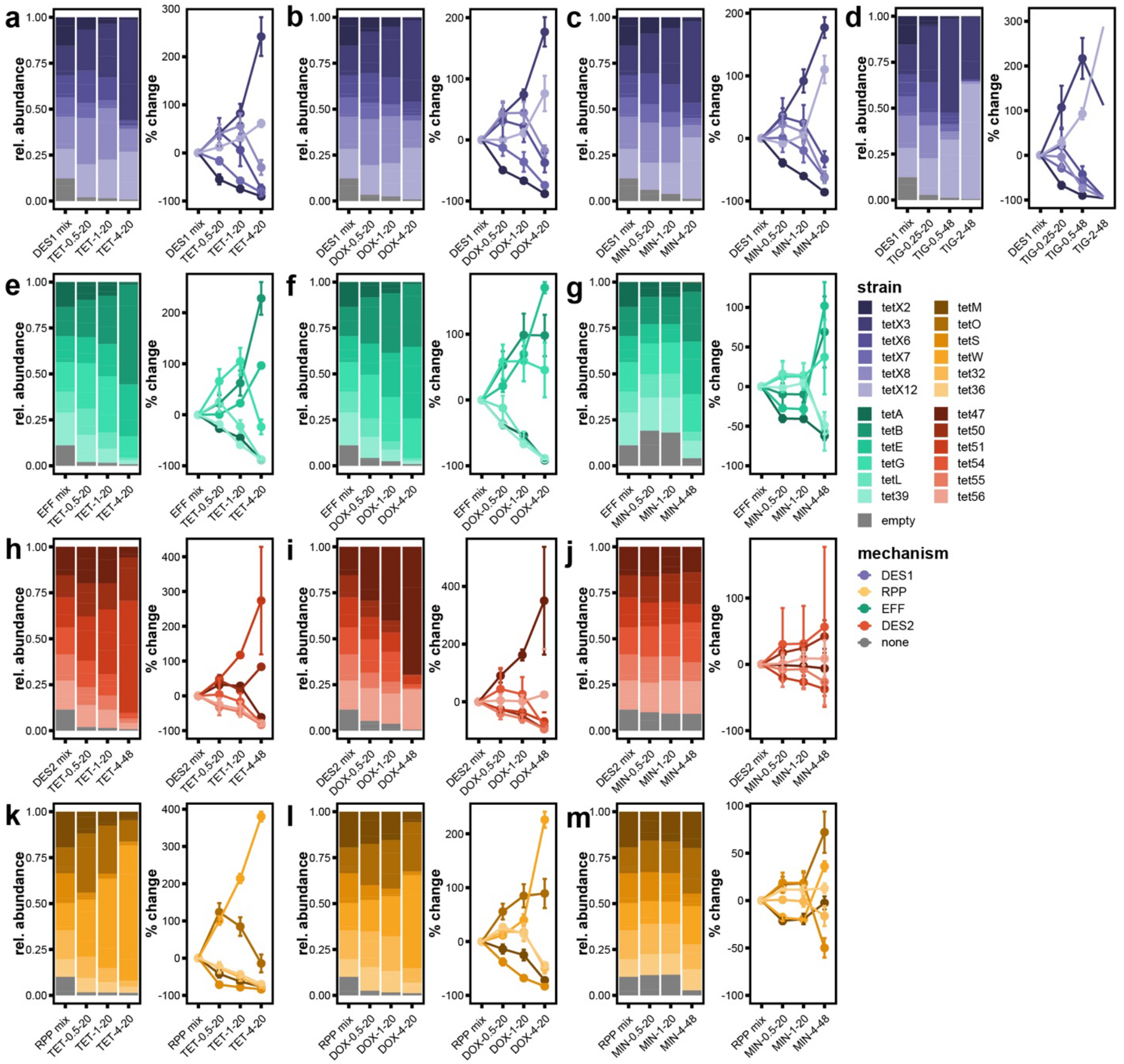


**Supplementary Figure 6.** Results for the mechanism-specific mixes. **(a-d)** DES1 with tetracycline, doxycycline, minocycline, and tigecycline. (**e-g)** EFF with tetracycline, doxycycline, and minocycline. **(h-j)** DES2 with tetracycline, doxycycline, and minocycline. **(k-m)** RPP with tetracycline, doxycycline, and minocycline. Panels D, E, and L also appear in Figure 4. X-axis labels denote antibiotic-concentration-timepoint (hours). Left panel: barplots of the relative abundance of each strain in the starting mix, then after growth in the antibiotic selective conditions at 0.5x, 1x, and 4x the MIC of the empty vector strain. Bar height represents the average of three replicates. Right panel: the percent change in the relative abundance of each strain. Points represent the average of three replicates, and error bars represent standard deviation. Abbreviations: TET =

tetracycline; DOX = doxycycline; MIN = minocycline; TIG = tigecycline; EFF = efflux pump; RPP = ribosomal protection protein; DES1 = type 1 tetracycline destructase; DES2 = type 2 tetracycline destructase.

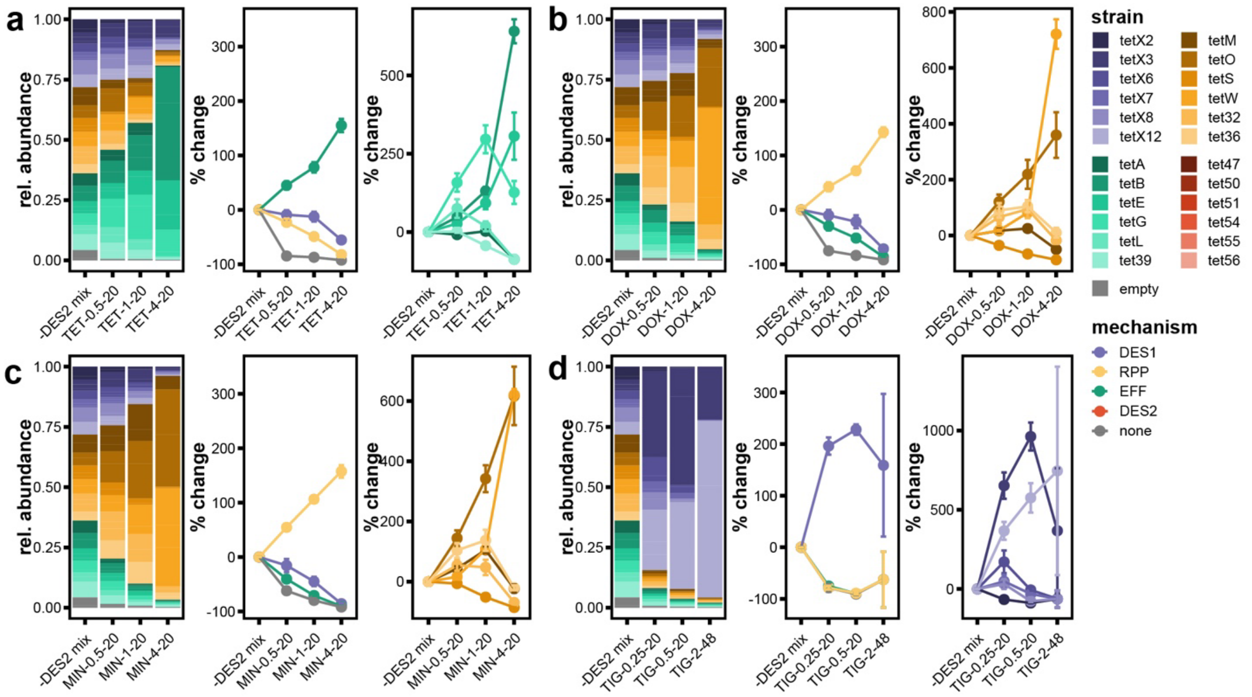


**Supplementary Figure 7.** Comparisons of strain relative abundance between the starting strain mixture containing all strains except those encoding DES2 genes (-DES2 mix) and the post-competition culture after 20-48 h growth at 0.5x, 1x, and 4x empty strain MIC for each antibiotic. Results for the -DES2 mix grown with **(a)** tetracycline, **(b)** doxycycline, **(c)** minocycline, and **(d)** tigecycline. X-axis labels denote antibiotic-concentration-timepoint (hours). Left panel: barplots of the relative abundance of each strain in the starting mix, then after growth in the antibiotic selective conditions at 0.5x, 1x, and 4x the MIC of the empty vector strain. Bar height represents the average of three replicates. Middle panel: the percent change in the summed relative abundances of each strain belonging to a given mechanism relative to the starting mixture. Right panel: the percent change in the relative abundance of each strain encoding genes belonging to the dominant mechanism under each antibiotic selective condition. Points represent the average of three replicates, and error bars represent standard deviation. Abbreviations: TET = tetracycline; DOX = doxycycline; MIN = minocycline; TIG = tigecycline; EFF = efflux pump; RPP = ribosomal protection protein; DES1 = type 1 tetracycline destructase; DES2 = type 2 tetracycline destructase.

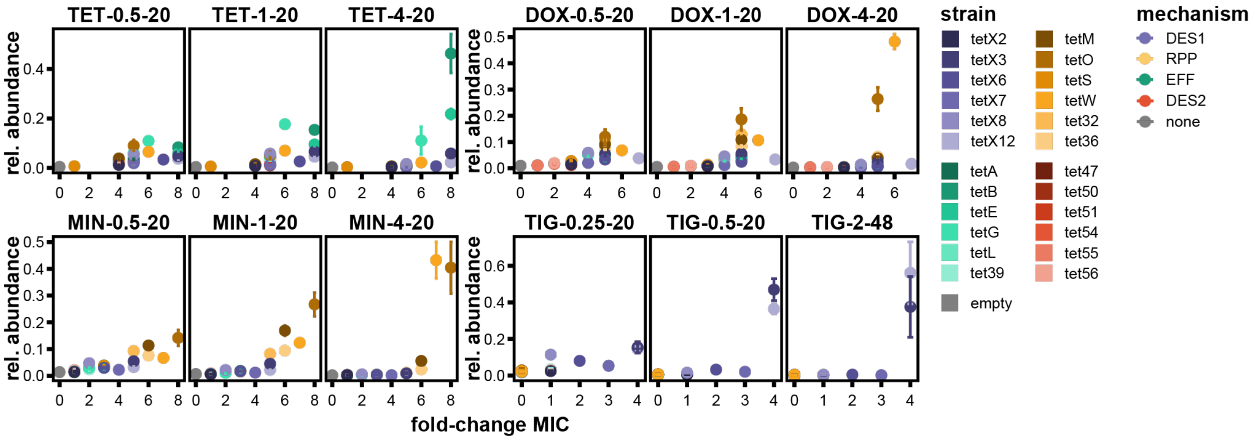


**Supplementary Figure 8.** Comparison of each strain's fold-change MIC to a given antibiotic, and strain relative abundance in the post-competition mix containing all 25 strains (ALL mix) grown after 20-48 h growth at 0.5x, 1x, and 4x empty strain MIC for that antibiotic. Resulting MIC values for each strain-antibiotic combination reported as log2 foldchange over the empty vector strain's MIC for that antibiotic. Plot labels denote antibiotic-concentration-timepoint (hours). Points represent the average of three replicates, and error bars represent standard deviation. Abbreviations: TET = tetracycline; DOX = doxycycline; MIN = minocycline; TIG = tigecycline; EFF = efflux pump; RPP = ribosomal protection protein; DES1 = type 1 tetracycline destructase; DES2 = type 2 tetracycline destructase.

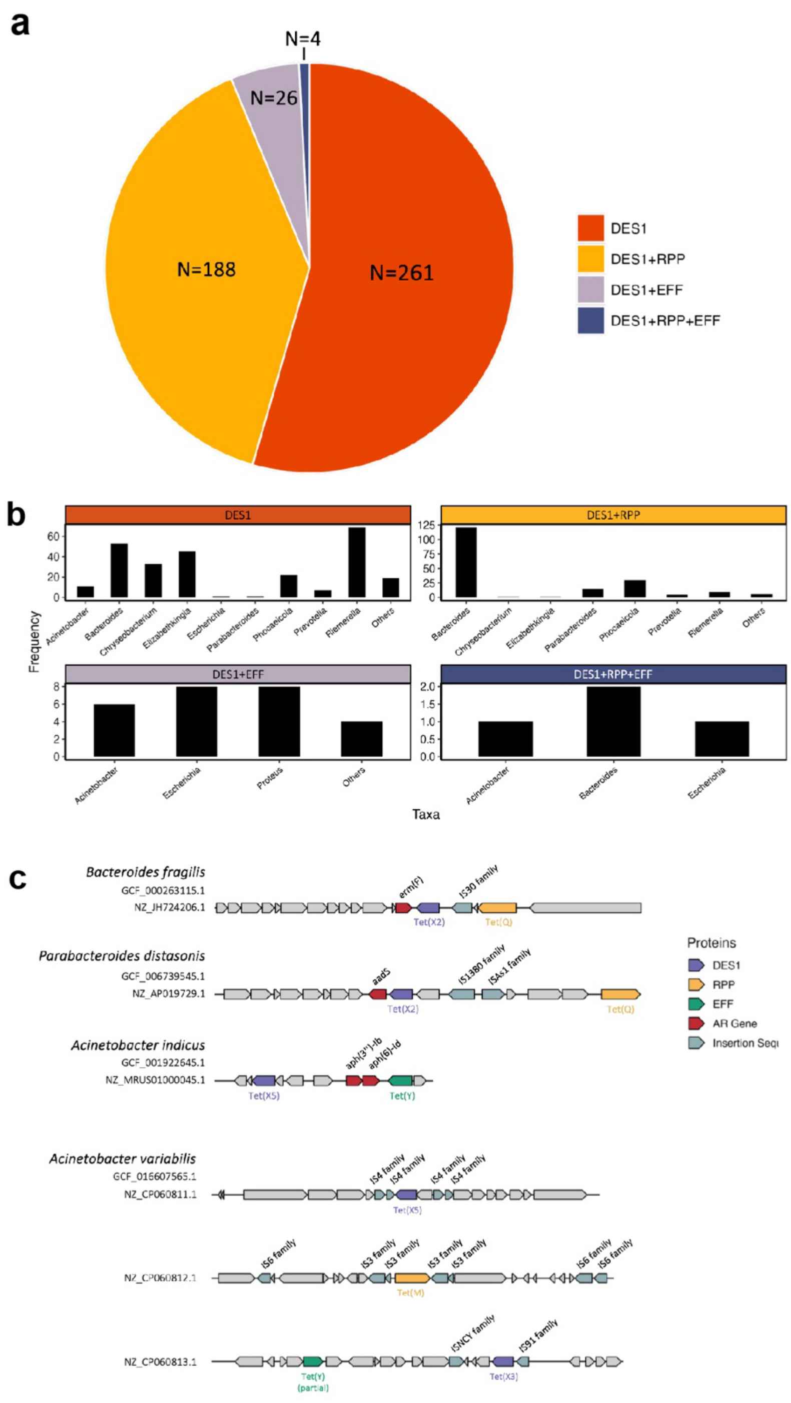


**Supplementary Figure 9.** Co-occurrence of tetracycline resistance genes. **(a)** Breakdown of 479 DES1-containing bacterial genome assemblies by different categories of tetracycline resistance gene co-occurrence. **(b)** Number of genomes per category further broken down by the number of taxa encoding those combinations. **(c)** Specific examples of mechanism co-occurrence. Lines represent contigs, bars represent ORFs, and bars are colored by gene type.

## Supplementary References


1. Blake, K.S. *et al.* Sequence-structure-function characterization of the emerging tetracycline destructase family of antibiotic resistance enzymes. *Commun Biol* **7**, 336 (2024).
2. Lutz, R. & Bujard, H. Independent and tight regulation of transcriptional units in Escherichia coli via the LacR/O, the TetR/O and AraC/I1-I2 regulatory elements. *Nucleic Acids Res* **25**, 1203-10 (1997).
