# Supplementary material for: The tetracycline resistome is shaped by selection for specific resistance mechanisms by each antibiotic generation
Source: Nat Commun. 2025 Feb 7;16:1452. doi: 10.1038/s41467-025-56425-5 (PMC11806011; doi:10.1038/s41467-025-56425-5)
Supplement: Supplementary file 2 — Description of Additional Supplementary Files [file 41467_2025_56425_MOESM2_ESM.pdf]

## Description of Additional Supplementary Files:

**Supplementary Data 1:** Tetracycline resistance genes used.

**Supplementary Data 2:** Genera in which tet resistance genes were identified.

**Supplementary Data 3:** AST results.

**Supplementary Data 4:** List of gene- and sample-specific barcodes.

**Supplementary Data 5:** Data for Supp. Fig. 9.

**Supplementary Data 6:** Primer sequences.

**Supplementary Data 7:** Strain mixture compositions.
